# Clinical Findings and Evaluation of Newborns Who Were Anonymously Surrendered

**DOI:** 10.1001/jamanetworkopen.2023.49853

**Published:** 2024-01-02

**Authors:** Claire D. Liepmann, Amira A. Nafiseh, James G. Carlucci, Leslie A. Enane

**Affiliations:** 1 The Ryan White Center for Pediatric Infectious Diseases and Global Health, Department of Pediatrics, Indiana University School of Medicine, Indianapolis; 2 Indiana University School of Medicine, Indianapolis

## Abstract

This cohort study examines clinical findings, medical treatment, and outcomes for infants in Indiana who were surrendered under Safe Haven laws.

## Introduction

All US states have Safe Haven laws, which legalize the anonymous surrender of newborns who may otherwise be at risk of abandonment. In an increasing number of states, Safe Haven Baby Boxes (SHBBs) provide a means for safe surrender of infants at fire stations and other locations. The number of SHBBs is the highest in Indiana.^1^ Clinicians evaluating infants who were surrendered are tasked with medical decision-making in the absence of maternal history or testing or knowledge of perinatal events. For example, there is a narrow window of time to intervene to prevent HIV in the infant when considering possible perinatal HIV exposure. In Indiana, the number of infants who were surrendered has been increasing. It is not yet known whether a state ban on most abortion procedures, which went into effect in August 2023, could result in further increases. The growing frequency of this clinical challenge prompted our group of pediatric infectious disease specialists to examine this cohort as we developed a harmonized clinical approach. We conducted a retrospective cohort study of infants surrendered under the Indiana Safe Haven law to determine the clinical findings, medical treatment, and outcomes in this vulnerable group.

## Methods

This cohort study followed the Strengthening the Reporting of Observational Studies in Epidemiology (STROBE) reporting guideline and was deemed exempt by the institutional review board at Indiana University. Informed consent was waived because the study was deemed minimal risk.

We searched electronic medical records to identify infants who were surrendered from January 1, 2013, to August 4, 2023. Two independent reviewers assessed records of infants (who were younger than 60 days) with hospital evaluation in the Indiana University (IU) Health system, HIV testing, and social work consult. Infants who were anonymously surrendered were included. Identified infants were cross-checked with those already known to our service. Infants with known maternal history or testing were excluded. Discordant or unclear determinations were adjudicated by a third reviewer. Clinical data regarding evaluations for perinatal infectious exposures or diagnoses were abstracted and described. Descriptive statistics were analyzed in Stata SE version 18.0 (StataCorp), and analysis was conducted from September 15 to November 13, 2023.

## Results

We reviewed records for 222 infants and identified 13 infants who were surrendered (7 male [54%] and 6 female [46%]) (Table). Most were estimated to be aged younger than 1 day, born term or late preterm, and presumably born outside of the health care setting. Among 13 infants, 12 (92%) were estimated to be 37 weeks’ gestation or later, and 1 (8%) was estimated to be 35 weeks’ gestation, or late preterm. Eleven infants (85%) were estimated to be aged 0 days, 1 infant (8%) was estimated to be aged 1 day, and 1 infant (8%) was estimated to be aged 5 days. Thirteen infants (100%) were tested for drug exposure and 12 (92%) were positive. Ten infants (77%) received antiretroviral medications, either until HIV testing returned (5 [39%]), or as a 4- to 6-week antiretroviral therapy (ART) regimen for possible high-risk HIV exposure was completed (5 [39%]). Evaluations at presentation and over subsequent months did not identify perinatal infections during the available follow-up time. We developed an institutional practice guideline based on key considerations (Box).

**Table.** Clinical Findings and Treatment of Infants Who Were Surrendered

Variables	Infants, No. (%) (N = 13)
Location of infant surrender	
Safe Haven Baby Box	8 (62)
Hospital	4 (31)
Fire station (without Safe Haven Baby Box)	1 (8)
Admitted to neonatal intensive care unit	11 (85)
Estimated age at presentation, median (range), d	0 (0 to 5)
Estimated gestational age, median (IQR), wk	40 (38 to 40)
Sex	
Male	7 (54)
Female	6 (46)
WHO weight-for-age *Z *score, median (IQR)	−0.6 (−0.8 to 0)
WHO length-for-age *Z *score, median (IQR)	0.4 (−0.3 to 0.5)
Drug testing (n = 13)^a^	
Positive for any substance	12 (92)
Positive for multiple substances	8 (62)
Amphetamine or methamphetamine	3 (23)
Barbiturate or benzodiazepine	2 (15)
Marijuana	10 (77)
Cocaine	4 (31)
Opiate or buprenorphine	5 (38)
Evaluations for perinatal exposure or infection	
HIV testing	
HIV-1/-2 antibody/antigen positive (n = 13)	0
HIV molecular test (viral load or qualitative PCR) positive (n = 8)	1 (13)^b^
No. of HIV molecular tests, median (IQR) (n = 8)	4.5 (1 to 5)
Age at last HIV molecular test, median (IQR), months (n = 8)	4.1 (1.3 to 4.5)
Hepatitis B virus testing	
Hepatitis B core antibody positive (n = 7)	0
Hepatitis B surface antigen positive (n = 4)	1 (25)^c^
Hepatitis B PCR positive (n = 3)	0
Hepatitis C virus testing	
Hepatitis C antibody positive (n = 9)	0
Hepatitis C PCR positive (n = 4)	0
Syphilis (treponemal testing) positive (n = 13)	0
Herpes simplex virus testing positive (n = 7)^d^	0
Hypothermia (<36.5 °C) within 6 h of presentation^e^	7 (54)
Blood culture positive (n = 12)	0
Elevated liver transaminases >2 × upper limit of normal (n = 8)	2 (25)
Abnormal chest radiography (n = 5)^f^	4 (80)
Abnormal head imaging (n = 2)^g^	0
Abnormal echocardiogram (n = 1)^h^	1 (100)
Therapies given	
Hepatitis B immune globulin	13 (100)
Hepatitis B vaccination	13 (100)
Vitamin K	13 (100)
Erythromycin eye ointment	13 (100)
Tetanus immune globulin	4 (31)
Antibiotic and antiviral therapies	
Any systemic antibiotic or antiviral	10 (77)
Penicillin single dose^i^	1 (8)
Ampicillin	9 (69)
Gentamicin	7 (54)
Third- or fourth-generation cephalosporin	6 (46)
Azithromycin	1 (8)
Acyclovir	5 (39)
Antiretroviral medications	
Any antiretrovirals for HIV	10 (77)
Antiretrovirals while awaiting initial HIV testing results only	5 (39)
Presumptive antiretroviral therapy regimen^j^	5 (39)
Age at last follow-up in Indiana University Health system, median (IQR) [range], months	4.1 (2.0 to 4.6) [0 to 75.9]

Abbreviations: PCR, polymerase chain reaction; WHO, World Health Organization.

^a^ Drug exposure testing from infant urine (12 infants), meconium (11 infants), and/or cord blood (4 infants). Multiple results may apply to an individual infant.

^b^ One infant who was not receiving antiretroviral medications had a positive HIV PCR below the limit of quantification; this was repeated several times and repeatedly negative. The initial positive result was interpreted as a false positive by clinicians.

^c^ Positive hepatitis B virus (HBV) surface antigen in 1 infant was interpreted by clinicians as likely false positive given recent HBV vaccination and negative HBV PCR.

^d^ Herpes simplex virus was tested using serum PCR (7 infants), cerebral spinal fluid PCR (1 infant), surface swab PCR (6 infants), and surface swab viral culture (1 infant).

^e^ Hypothermia defined as a temperature less than 36.5 °C, per the WHO. Of these 7 infants, 5 infants presented hypothermic but had normal temperatures within 2 hours; 1 developed hypothermia that resolved within 4 hours; and 1 infant was hypothermic and critically ill on presentation, with issues including seizures, respiratory failure, and hypoxic ischemic encephalopathy.

^f^ Chest radiography abnormalities include interstitial (1 infant), perihilar (2 infants), and diffuse (1 infant) opacities, and small pleural effusions (1 infant).

^g^ Two infants had head ultrasonography, and 1 of these infants had brain magnetic resonance imaging.

^h^ Echocardiogram was performed for murmur; identified atrial septal defect.

^i^ Dosing for possible congenital syphilis. Treponemal testing later resulted negative in this instance.

^j^ Presumptive antiretroviral therapy regimens include 3 antiretroviral medications at treatment doses per US federal guidelines given for 4 weeks (1 infant) or 6 weeks (4 infants).^2^

Box. **Considerations for the Infectious Evaluation of Newborns Who Were Anonymously Surrendered^a^**

**General considerations**

Clinicians should always consult with pediatric infectious disease experts in their setting regarding the evaluation and management of infants who were anonymously surrendered or abandoned
The infant antibody tests described later are used when the mother is not present for evaluation, as proxy evaluations for maternal antibody status and perinatal exposure
Infant antibody testing has the important limitation that results may be falsely negative in the setting of acute or recent maternal acquisition of infection prior to delivery. Therefore, additional follow-up testing is warranted to evaluate for perinatal infection as described under each heading
Positive results require further evaluation and management for possible perinatal exposure or infection in consultation with the pediatric infectious disease consultant
Relevant current clinical guidelines should be reviewed directly, including perinatal HIV guidance from the US Department of Health and Human Services^2^ or other national guidelines if outside the US, as well as national guidance for pediatric infectious diseases, such as the American Academy of Pediatrics *Red Book Report of the Committee on Infectious Diseases*^3^

**HIV**

Rapid HIV antibody or expedited fourth generation HIV antibody–antigen test should be performed to evaluate maternal antibody as an indicator of perinatal HIV exposure^4^
HIV RNA PCR should be performed at presentation to evaluate for established or in utero HIV infection
If HIV RNA PCR and HIV antibody (or antibody–antigen) are negative at presentation, repeat HIV RNA PCR at age 1 to 2 months and again at age 4 to 6 months. Two negative molecular HIV tests at age 1 month or older and at 4 months or older confirm HIV-negative status in infants who are not breastfed^2^
If testing demonstrates evidence of perinatal HIV exposure or infection, rapid initiation of antiretroviral medications and confirmatory diagnostic testing should follow US federal guidelines^2^ and pediatric HIV specialist consultation
If HIV antibody and RNA PCR negative, presumptive antiretroviral therapy is generally not indicated, but may be considered depending on local HIV prevalence and/or acute risk for recent maternal HIV acquisition (eg, infant screen positive for opiate exposure)

**HBV**

All infants should receive HBV immunoglobulin and vaccination as soon as possible.^a,b^ Administration of these preventive interventions should not be delayed while obtaining laboratory evaluations
HBV core and HBV surface antibody may be useful (to evaluate maternal antibody), if collected before HBV vaccination and immunoglobulin are administered.^c^ HBV surface antigen may be considered (to evaluate infant infection), but may be low-yield if obtained soon after birth
HBV surface antibody and HBV surface antigen may be evaluated at 9 to 12 months of age after completion of HBV vaccination series

**HCV**

HCV antibody (to evaluate maternal antibody) should be tested at presentation^d^
HCV RNA PCR should be tested at 2 to 6 months of age^5^

**HSV**

Evaluation and management for neonatal HSV will depend on clinical findings^a^

**Syphilis**

All infants should be screened with RPR
If RPR is negative, repeat at 3 months of age
Positive testing and/or abnormal physical examination should prompt complete evaluation and management for congenital syphilis^a^

**Tetanus**

Tetanus immunoglobulin is indicated if umbilical cord is grossly contaminated^a,e^

**Sepsis**

Evaluation and management for neonatal sepsis including consideration of antimicrobial therapy will depend on clinical findings^f^

**Additional evaluations and management**

All infants should be evaluated for perinatal drug exposures
Intramuscular vitamin K is indicated (if infant age estimated younger than 2 weeks)
Erythromycin ophthalmic ointment is indicated (if infant age estimated younger than 48 hours)
Ongoing follow-up is recommended with pediatric infectious disease consultants for further testing in the infant period
Ongoing medical and developmental evaluations in pediatric medical home

Abbreviations: HBV, hepatitis B virus; HCV, hepatitis C virus; HSV, herpes simplex virus; PCR, polymerase chain reaction; RPR, rapid plasma reagin.

^a^
Further testing and recommendations as per American Academy of Pediatrics Red Book^3^ and pediatric infectious disease consultation.

^b^
Hepatitis B immunoglobulin is unlikely to be effective for perinatal HBV prevention after 7 days of life.^3^

^c^
Presence of HBV core antibody could represent either previous (cleared or treated) or active maternal HBV infection. Presence of maternal HBV surface antibody indicates either vaccination or resolution of past infection, signaling that the infant is likely not exposed to HBV.

^d^
Presence of HCV antibody could represent either previous (cleared or treated) or active maternal HCV infection.

^e^
If tetanus immune globulin is not available, intravenous immunoglobulin may be used.^3^ Tetanus vaccine administration is not appropriate in the neonatal period; it should be given according to the current federal vaccination schedule.

^f^
Further testing and recommendations as per neonatal or pediatric expertise and pediatric infectious disease consultation.

## Discussion

No clinical guidelines exist for the treatment of infants who were surrendered, and perinatal infections affecting this medically and socially vulnerable population have not been characterized. In this cohort study, we present findings from a US state with poor maternal and infant outcomes, an opioid epidemic, and the longest-running SHBB program. Our findings highlight prevalent drug exposures, signaling acute potential risk for perinatal infections, in addition to other medical, developmental, and behavioral outcomes. While we did not identify HIV exposure or perinatal infections, the small cohort size and variable practice patterns do not allow for generalizable estimates of these risks. This study is further limited by its retrospective nature and restriction to IU Health.

Substantial practice variations in this cohort demonstrate the need for a harmonized clinical approach (Box). Expedited infant testing for HIV antibodies indicates maternal antibody status and perinatal HIV exposure,^4^ while HIV RNA polymerase chain reaction (PCR) tests for HIV infection. Repeated PCR testing over time is warranted, given possible recent antenatal maternal HIV infection not evidenced by antibody response. Presumptive ART is initiated if testing suggests perinatal HIV exposure or infection while results are confirmed.^2^ The approach may differ in settings with high HIV prevalence. Further research is needed regarding the management and perinatal and longer-term outcomes of infants who were surrendered anonymously.

## Notes

Supplement.
Data Sharing Statement

## References

[1] Baby Box Locations. Safe Haven Baby Boxes. Accessed September 20, 2023. https://shbb.org/location

[2] Panel on Treatment of HIV During Pregnancy and Prevention of Perinatal Transmission. Recommendations for the use of antiretroviral drugs during pregnancy and interventions to reduce perinatal HIV transmission in the United States. HIV.gov. Accessed September 18, 2023. https://clinicalinfo.hiv.gov/en/guidelines/perinatal/recommendations-arv-drugs-pregnancy-overview

[3] American Academy of Pediatrics. Red Book: 2021 Report of the Committee on Infectious Diseases. 32nd ed. American Academy of Pediatrics; 2021.

[4] American Academy of Pediatrics Committee on Pediatric AIDS. HIV testing and prophylaxis to prevent mother-to-child transmission in the United States. Pediatrics. 2008;122(5):1127-1134. doi:10.1542/peds.2008-2175 18977995

[5] Panagiotakopoulos L, Sandul AL, Conners EE, Foster MA, Nelson NP, Wester C; DHSc; Collaborators. CDC recommendations for Hepatitis C testing among perinatally exposed infants and children—United States, 2023. MMWR Recomm Rep. 2023;72(4):1-21. doi:10.15585/mmwr.rr7204a1 37906518 PMC10683764

