# Supplementary material for: Clinical Findings and Evaluation of Newborns Who Were Anonymously Surrendered
Source: JAMA Netw Open. 2024 Jan 2;7(1):e2349853. doi: 10.1001/jamanetworkopen.2023.49853 (PMC10762573; doi:10.1001/jamanetworkopen.2023.49853)
Supplement: Supplement. — Data Sharing Statement [file jamanetwopen-e2349853-s001.pdf]

Data Sharing Statement

Liepmann. Clinical Findings and Evaluation of Newborns Who Were Anonymously Surrendered. *JAMA Netw Open*. Published January 02, 2024. doi:10.1001/jamanetworkopen.2023.49853

Data
Data available: No

Additional Information
Explanation for why data not available: We are unable to share individual data given ethical and privacy concerns. Specifically, given this small and unique cohort there may be potential for identification based on limited characteristics.
